# Supplementary material for: Novel Auger-Electron-Emitting 191Pt-Labeled Pyrrole–Imidazole Polyamide Targeting MYCN Increases Cytotoxicity and Cytosolic dsDNA Granules in MYCN-Amplified Neuroblastoma
Source: Pharmaceuticals (Basel). 2023 Oct 27;16(11):1526. doi: 10.3390/ph16111526 (PMC10675227; doi:10.3390/ph16111526)
Supplement: Supplementary file 1 [file pharmaceuticals-16-01526-s001.zip › pharmaceuticals-2631815-supplementary.pdf]

Supplementary Information

Novel Auger-Electron-Emitting ^{191}Pt-Labeled Pyrrole–Imidazole Polyamide Targeting MYCN Increases Cytotoxicity and Cytosolic dsDNA Granules in MYCN-Amplified Neuroblastoma

Honoka Obata [1,2,3], Atsushi B. Tsuji [2,*], Hitomi Sudo [2], Aya Sugyo [2], Kaori Hashiya [4], Hayato Ikeda [5,6], Masatoshi Itoh [5], Katsuyuki Minegishi [1], Kotaro Nagatsu [1], Mikako Ogawa [3], Toshikazu Bando [4], Hiroshi Sugiyama [7] and Ming-Rong Zhang [1]

[1] Department of Advanced Nuclear Medicine Sciences, National Institutes for Quantum Science and Technology (QST), 4-9-1 Anagawa, Inage-ku, Chiba 263-8555, Japan
[2] Department of Molecular Imaging and Theranostics, National Institutes for Quantum Science and Technology (QST), 4-9-1 Anagawa, Inage-ku, Chiba 263-8555, Japan
[3] Graduate School of Pharmaceutical Sciences, Hokkaido University, Kita-ku, Sapporo, Hokkaido 060-0812, Japan.
[4] Department of Chemistry, Graduate School of Science, Kyoto University, Kitashirakawa-oiwakecho, Sakyo-ku, Kyoto 606-8502, Japan
[5] Cyclotron and Radioisotope Center (CYRIC), Tohoku University, Sendai, Miyagi 980-8578, Japan
[6] Research Center for Electron Photon Science (ELPH), Tohoku University, Sendai, Miyagi 982-0826, Japan
[7] Institute for Integrated Cell-Material Science (iCeMS), Kyoto University, Yoshida-ushinomiyacho, Sakyo-ku, Kyoto 606-8501, Japan.
* Correspondence: tsuji.atsushi@qst.go.jp; Tel.: +81-43-382-3704

Table of Contents

Supplementary Materials and Methods 1
Supplementary Figures 3
Supplementary Figure S.1 3
Supplementary Figure S.2 3
Supplementary Figure S.3 4
Supplementary Figure S.4 4
Supplementary Table S.1 5

Supplementary Materials and Methods

Reagents

Fmoc-Py-COOH (FUJIFILM Wako Pure Chemical, Osaka, Japan) [CAS195387-29-2], Fmoc-Im-COOH (FUJIFILM Wako Pure Chemical) [CAS 252206-28-3], Fmoc-β-alanine (Novabiochem; Merck, Darmstadt, Germany) [CAS 35737-10-1], Fmoc-γ-abu-COOH (Watanabe Chemical Industries, Hiroshima, Japan) [CAS 116821-47-7], Fmoc-PyIm-COOH (FUJIFILM Wako Pure Chemical) [CAS 1040393-13-2], Fmoc-Cys(Trt)-OH (Peptide Institute, Osaka, Japan) [CAS 103213-32-7], Fmoc-D-Arg(Pbf)-OH (Watanabe Chemical Industries) [CAS 1217461-89-6].

Production of ^{191}Pt

^{191}Pt was produced via the ^{nat}Ir(p, xn)^{191}Pt reaction using a 30 MeV proton beam for 7 h at a beam current of 2–10 μA; the experiments were conducted at the AVF-930 cyclotron at the National Institutes for Quantum Science and Technology (QST)

or the cyclotron at the Tohoku University Cyclotron and Radioisotope Center (CYRIC). After several days of cooling time, n.c.a. ^{191}Pt was separated from an Ir target material as described previously [36,37,11]. The obtained ^{191}Pt aqueous solution contained KCl (0.2–0.4 mol/L) and phosphate ion (0.2 mol/L), and the concentration of ^{191}Pt activity was 25–620 kBq/μL. Most of the n.c.a. ^{191}Pt was $^{191}Pt^{II}Cl_4^{2-}$ (60–80%); some of it was hydrolyzed to neutral complexes such as $[^{191}Pt^{II}Cl_{4-n}(H_2O)_n]^{(2-n)-}$. Hydrolyzed Pt in the form $[PtCl_{4-n}(H_2O)_n]^{(2-n)-}$ is known to be reactive toward amines or sulfur-containing agents according to the mechanism of Pt-based drugs. Mixtures of $^{191}PtCl_4^{2-}$ and $[^{191}PtCl_{4-n}(H_2O)_n]^{(2-n)-}$ are hereafter referred to as "free ^{191}Pt"; they were used as the precursor in the present work as well as in our previous study [11]. Free ^{191}Pt was also used in in vitro experiments as a control and was prepared in a PBS solution containing 0.2% Tween 80.

Cell culture

The human neuroblastoma cell lines Kelly, SK-N-DZ, and SK-N-AS were obtained from ECACC (Salisbury, UK). The cells were cultured at 37°C in a humidified atmosphere containing 5% CO_2 in RPMI-1640 (FUJIFILM Wako Pure Chemical) containing 10% fetal bovine serum (Thermo Fisher Scientific, Waltham, MA, USA).

Fluorescent imaging of 53BP1-EGFP in U2OS-53BP1 cells

U2OS-53BP1 (U2RDP-LE53-21) cells stably expressing tumor-suppressor p53-binding protein 1 (53BP1) linked to a green fluorescent protein (EGFP) were established and provided by Dr. Kurimasa at Tohoku Medical and Pharmaceutical University. The cells were cultured at 37°C in a humidified atmosphere containing 5% CO_2 in RPMI-1640 (FUJIFILM Wako Pure Chemical) with 10% FBS (Thermo Fisher Scientific, Waltham, MA, USA). U2OS-53BP1 (6×10^5 cells/well, 1.5 mL media) cells were seeded onto a glass-based 6-well culture plate (EZVIEW® culture plates LB; AGC Techno Glass, Shizuoka, Japan) and incubated overnight at 37°C in a humidified atmosphere containing 5% CO_2. A solution containing nonradioactive MYCN-PIP (1.5 nmol) with or without ^{191}Pt-MYCN-PIP (290 kBq) was added to the cells. The cells were incubated in the medium containing each agent after 16 h of incubation, and the medium was replaced with fresh Opti-MEM™ (no phenol red; Thermo Fisher Scientific) containing 10% FBS. After 0 d and 1 d of incubation, fluorescence microphotographs for 53BP1-EGFP of the cells were acquired with an inverted fluorescence microscope (BZ-X800 fluorescence microscope; KEYENCE, Osaka, Japan). The fluorescence microscope was equipped with a 20× objective lens (Plan Apochromat; Nikon Solutions, Tokyo, Japan) and a fluorescent filter for EGFP (49020-UF1-BLA, excitation 480/20, emission 510/20; M SQUARE, Fukuoka, Japan). The quantitative analysis of images was performed using the "hybrid cell count" method of the software under the same conditions for each image. The nuclei and 53BP1-EGFP foci were extracted from the images semi-automatically and were used as the basis for the area calculation.

Animal studies on the xenografted tumor model

The protocol for the animal experiments was approved by the Animal Care and Use Committee of the National Institutes for Quantum and Radiological Science and Technology (13–1022, 26 May 2016), and all animal experiments were conducted following the institutional guidelines regarding animal care and handling. Kelly or SK-N-AS cells were suspended in a mixture of Matrigel and PBS (1:1), and 5×10^6 cells in 100 μL were subcutaneously injected into the flank of female BALB/c-nu/nu mice (4 weeks old for Kelly, 6 weeks old for SK-N-AS; CLEA Japan, Tokyo, Japan) under isoflurane anesthesia. Tumor volumes reached approximately 100–500 mm^3 after 3–4 weeks for Kelly and 2–3 weeks for SK-N-AS after injection.

For the biodistribution study, ^{191}Pt-MYCN-PIP (168–229 kBq, 0.1 nmol of nonradioactive MYCN-PIP) was intravenously or intratumorally injected into mice (Kelly and SK-N-AS) when tumor volumes reached approximately 100–500 mm^3 ($4 \leq n \leq 5$ for each time-point). The mice were sacrificed by isoflurane inhalation at 1, 2, 4, or 7 days after injection. Blood was obtained from the heart, and then tumor, lung, liver, spleen, intestine, kidney, bone, and muscle were dissected. The uptake is represented as a percentage of the injected dose (radioactivity) per gram of tissue (%ID/g).

Red blood cell partitioning and plasma protein binding

The red blood cell (RBC) partitioning and the plasma protein binding of ^{191}Pt were evaluated using the blood of mice. At 60 min, 1 d, and 4 d after intravenous injection of ^{191}Pt-MYCN-PIP (229 kBq, 0.1 nmol of nonradioactive MYCN-PIP), the blood was obtained from the heart and mixed with heparin (Mochida Pharmaceutical Factory, Tokyo, Japan). The whole blood was centrifuged and divided into RBCs and plasma. The RBC partitioning rate of ^{191}Pt was calculated on the basis of the activity of blood and plasma, defined as $(A_{blood} - A_{plasma}) \times (1 - Hct) \times 100/A_{blood}) \times 100$, where A_{blood} is the radioactivity of 20 μL whole blood, A_{plama} is the radioactivity of a 20 μL plasma fraction, and Hct is the hematocrit value (0.43). The plasma (50 μL) was put onto a spin column packed with Sephadex G-50 column (GE Healthcare, Chicago, IL, USA) eluted with 0.1 M sodium acetate solution. The protein binding rate of ^{191}Pt was calculated on the basis of the activity of the gel filtered fraction and the column, defined as $A_{sol}/(A_{col} + A_{sol}) \times 100$, where A_{col} is the radioactivity of a column and A_{sol} is the radioactivity of a filtered fraction.

DNA extraction from tumor tissue

Tumors were excised 10 days after injection of ^{191}Pt-MYCN-PIP. Twenty-five milligrams of tumor tissues were homogenized

in PBS using a homogenizer (BioMasher I; Nippi, Tokyo, Japan) and dissolved in a lysis buffer. The genomic DNA was isolated from tumor tissues under the same protocol used for the DNA-binding assay. The concentration of RNA and DNA was determined using a NanoDrop One microvolume UV–Vis spectrophotometer (Thermo Fisher Scientific).

References

36. Obata H, Minegishi K, Nagatsu K, Zhang MR, Shinohara A. Production of ^{191}Pt from an iridium target by vertical beam irradiation and simultaneous alkali fusion. *Appl Radiat Isot.* **2019**;149:31–37.
37. Obata H, Minegishi K, Nagatsu K, Ogawa M, Zhang MR. Synthesis of no-carrier-added [$^{188,\ 189,\ 191}$Pt]cisplatin from a cyclotron produced $^{188,\ 189,\ 191}PtCl_4{}^{2-}$ complex. *Sci Rep.* **2021**;11:8140.
11. Obata H, Tsuji AB, Kumata K, Sudo H, Minegishi K, Nagatsu K, Takakura H, Ogawa M, Kurimasa A, Zhang MR. Development of novel ^{191}Pt-labeled Hoechst33258: ^{191}Pt is more suitable than ^{111}In for targeting DNA. *J Med Chem.* **2022**;65:5690−5700

Supplementary Figures

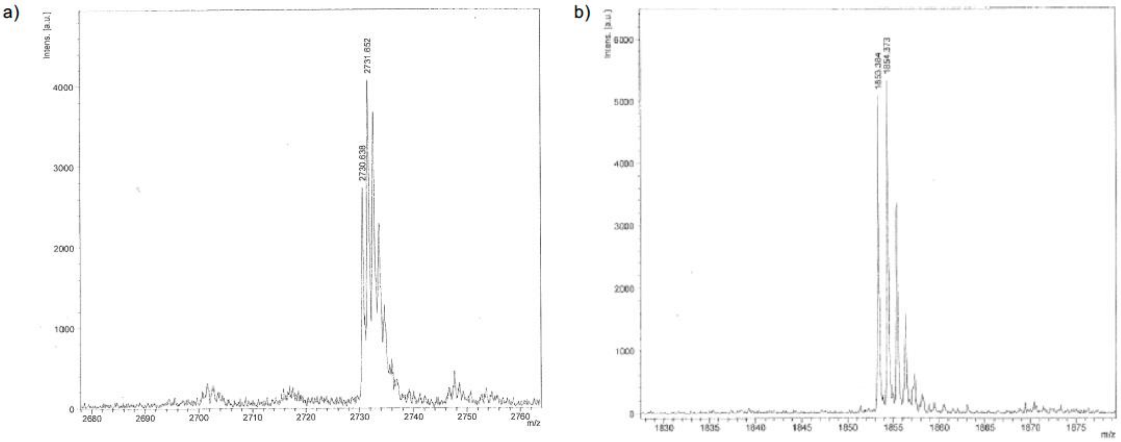

Figure S 1. MALDI-TOF MS spectra of (**a**) MYCN-PIP and (**b**) GCC-PIP.

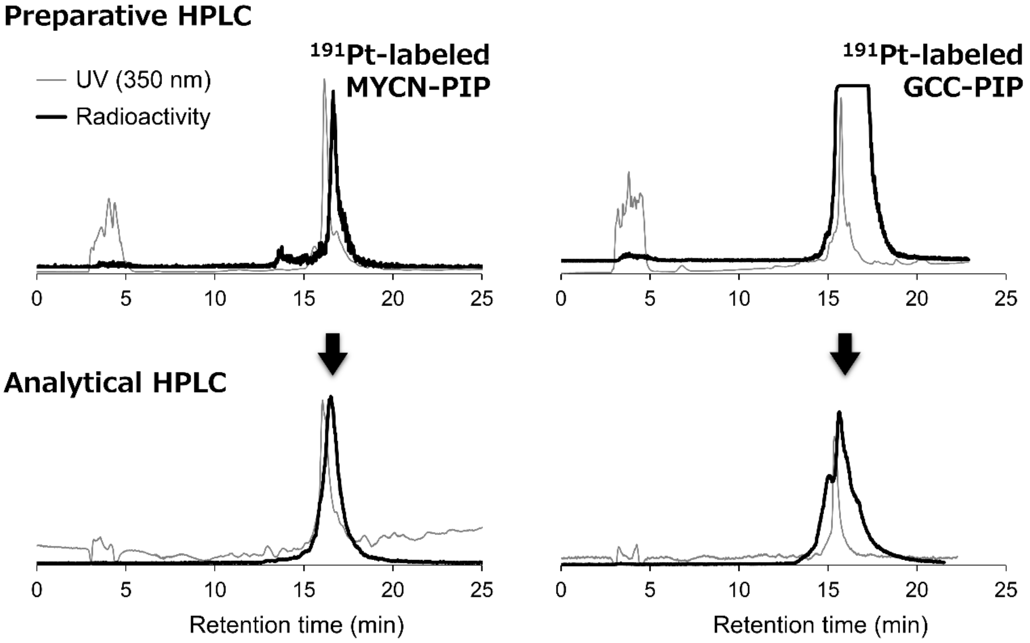

Figure S2. Radio chromatogram of preparative and analytical HPLC for the product of [^{191}Pt]Pt-MYCN-Cys-R3-coumarin (^{191}Pt-MYCN-PIP) and [^{191}Pt]Pt-GCC-Cys-R3-coumarin (^{191}Pt-GCC-PIP). HPLC conditions: column, COSMOSIL 5C18-MS-II; gradient elution, A/B = 0.1% trifluoroacetic acid (TFA) in H_2O/CH_3CN = 95/5 → 30/70 (25 min).

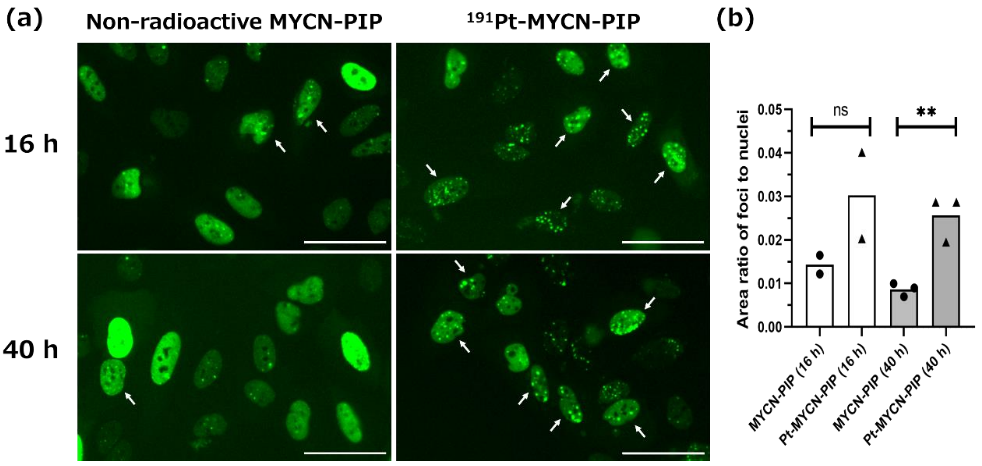

Figure S3. (**a**) Representative microphotographs of U2OS-53BP1 cells 16 and 40 h after the treatment with nonradioactive MYCN-PIP (1.5 nmol) with or without ^{191}Pt-MYCN-PIP (290 kBq). Signals of 53BP1-EGFP are indicated in green. Objective lens: 20×; scale bars: 50 µm. Arrows show the cells with 53BP1 foci. (**b**) Area ratio of 53BP1 foci to nuclei. Data were obtained from five different images with 18–94 nuclei per image. Each dot indicates a value per image. The data were evaluated by *t*-test using the GraphPad Prism 9 software (GraphPad Software, San Diego, CA, USA); ns: $p \geq 0.05$,** $p < 0.01$. $p < 0.05$ was considered statistically significant. Each dot indicates a value per image.

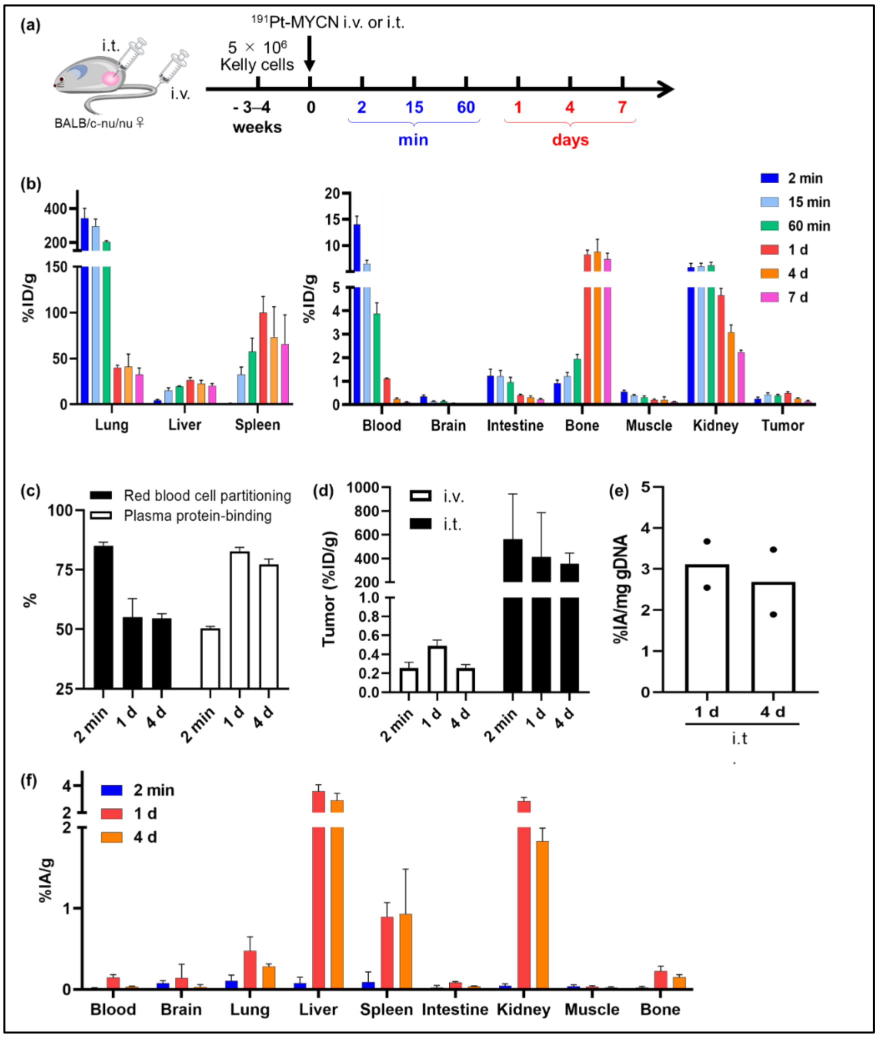

Figure S4. (**a**) Experimental schedule of in vivo experiments using mice bearing a Kelly tumor to evaluate the biodistribution of ^{191}Pt-MYCN-PIP, where intravenous (i.v.) and intratumoral (i.t.) injections are compared (^{191}Pt-MYCN-PIP: 168–229 kBq; nonradioactive MYCN-PIP: 0.1 nmol, 5

nmol/kg body weight) (corrected to 20 g body weight, n = 4–5). (**b**) Biodistribution of ^{191}Pt-MYCN-PIP at 2 min to 7 d after intravenous injection (i.v.) in mice bearing a Kelly tumor (n = 5). (**c**) Red blood cell partitioning rate (%) and plasma protein-binding rate (%) (n = 4). (**d**) Percentage of the injected activity per 1 g of the Kelly tumors (%IA/g) (corrected to 20 g body weight, n = 4–5). (**e**) DNA-binding fraction of ^{191}Pt per mg of tumor gDNA (%IA/mg gDNA) (n = 2). (**f**) Biodistribution of ^{191}Pt-MYCN-PIP at 2 min, 1 d, and 4 d after intratumoral injection in mice bearing a Kelly tumor (n = 4).

Table S1. DNA-binding rate (%ID/mg DNA) and the ratio of DNA binding to cell fraction (%) of ^{191}Pt in Kelly cells at 1 d after the incubation or injection of ^{191}Pt-MYCN-PIP.

	***In vitro* (*n* = 3)**	***In vivo* (*n* = 2)**
Cell or tumor fraction (%ID)	24 ± 1	56; 64
DNA-binding/cell fraction (%)	0.38 ± 0.04	0.30; 0.46
DNA-binding rate (%ID/mg gDNA)	41 ± 3	3.7; 2.5
DNA volume per fraction (µg)	2.2 ± 0.4	17; 34
